# A Possible Sensory Interpretation of Alternate Motor Fibers Relating to Structural Reserve during Stroke Recovery

**DOI:** 10.3389/fneur.2017.00355

**Published:** 2017-07-25

**Authors:** Sofya P. Kulikova, Vadim V. Nikulin, Larisa A. Dobrynina, Maria A. Nazarova

**Affiliations:** ^1^National Research University Higher School of Economics, Perm, Russia; ^2^Perm State University, Perm, Russia; ^3^Centre for Cognition and Decision Making, National Research University Higher School of Economics, Moscow, Russia; ^4^Department of Neurology, Max Planck Institute for Human Cognitive and Brain Sciences, Leipzig, Germany; ^5^Research Center of Neurology, Moscow, Russia

**Keywords:** diffusion-weighted magnetic resonance imaging, stroke, brain structural reserve, motor recovery, alternate motor fibers, corticospinal tract, spinothalamic tract, sensory pathways

Recovery after stroke relates tightly to the white matter integrity. Currently, the main methodology for non-invasive white matter integrity assessment is diffusion-weighted magnetic resonance imaging (DW-MRI), a state-of-the-art approach which is, however, prone to multiple limitations (1). Using DW-MRI, it was demonstrated that many pathways including corticospinal tract (CST) and corpus callosum contribute to structural brain reserve (2) after stroke, but only a few of these tracts were found to be useful in the clinical practice. The most widely known measure is an asymmetry of the fractional anisotropy (FA) in CST at the level of the internal capsule, which could be used for predicting motor recovery in acute stroke (3). Recently, a new complementary motor component of the structural reserve, the so-called alternate motor fibers (AMFs), was proposed for motor recovery prognosis in stroke patients (4), and it was even reported to correlate with the effect of the transcranial direct current stimulation in chronic stroke (5). Here, we would like to point out a possible additional sensory interpretation of the AMF that appears plausible after taking into account technical limitations of DW-MRI approach, which may potentially give rise to different interpretations of the same results.

Alternate motor fibers, introduced by Lindenberg et al. (4), were defined as the set of fibers resulting from deterministic tractography based on the diffusion tensor model and three regions of interest (ROIs): (1) the precentral gyrus and its underlying white matter; (2) the posterior limb of the internal capsule (PLIC); and (3) the pontine ROI just below the level of the superior cerebellar peduncles. These fibers appeared in addition to CST fibers reconstructed using the same ROIs in the precentral gyrus and PLIC with a narrow pontine ROI containing only posterior part of the pons (tegmentum pontis).

Probably, this made the authors consider AMFs as primarily descending motor pathways comprising, for example, the cortico-rubro-spinal tract (6). However, such reconstruction also warrants a possible sensory interpretation of AMF considering that small pontine ROI contains many densely packed ascending and descending tracts (7), while DW-MRI tractography cannot differentiate ascending and descending fibers (1).

Indeed, selection of ROIs is not a standard procedure yet and presents one of the sources for potential bias in interpretation of the tractography results. Supporting the hypothesis of an additional sensory interpretation of AMF, comparison of the pontine ROI, used to capture the AMF, with the DTI atlas of Wakana et al. (7) indicates that it contains several white matter pathways, including dorsal and medial longitudinal fasciculi, the central tegmental tract, and medial lemniscus.

Other sources of ambiguity in interpretation of DW-MRI data are linked to the choice of the diffusion model and tractography algorithm. In the work by Lindenberg et al. (4), the diffusion tensor model (1) was used for AMF tractography, where white matter fibers are reconstructed by following the direction of the principal eigenvector of the diffusion tensor from one voxel to another. Being computationally simple, this method has several limitations. First, as tractography exploits only the principle eigenvector, it cannot resolve more than one fiber direction per voxel, unrealistically assuming a homogeneous unidirectional assembly of fibers within each voxel. Thus, in regions containing several fiber populations with complex architecture (for example, containing crossing or kissing fibers), this approach could give incorrect estimations of fiber directions. Using anisotropic voxels, as in the work by Lindenberg et al. (4, 8), may further bias both the estimation of fiber orientation and FA values. In case of anisotropic voxels, separate calculations along the section direction may be useful to estimate the orientation of the vector connecting the neighboring voxels.

Second, the diffusion tensor model may be not reliable in regions with low signal-to-noise ratio and/or FA values because of a large uncertainty in the principle eigenvector direction. In this case, fibers from different pathways with incidentally similar local orientations may be falsely grouped together by the tractography algorithm (1). For example, arcuate fasciculus and CST may be artificially combined in the region of their crossing, while in the temporal lobe the middle longitudinal fasciculus may result in false continuation of the arcuate fasciculus. Similar situations may potentially occur even in high FA areas where several pathways run in parallel and could not be discriminated due to a large voxel size or not accurate ROI placement.

It might be that in case of AMF we may encounter an analagous situation. To demonstrate this, we reconstructed AMF according to Lindenberg et al. (4) using demo-data from BrainVISA software (http://brainvisa.info/web/downloadpage_wrap.html) and compared them with spinothalamic tract (STT) fibers that we reconstructed from the same data using the strategy described by Dubois et al. (9) (Figure 1). One may notice that between the pons and the thalamus the reconstructions of AMF and STT appear quite similar. Similar fiber trajectories are also shown for STT in other studies, as well as for other sensory pathways, for example, for medial lemniscus fibers (7, 9, 10), pointing out the difficulties in their separation.

**Figure 1:**
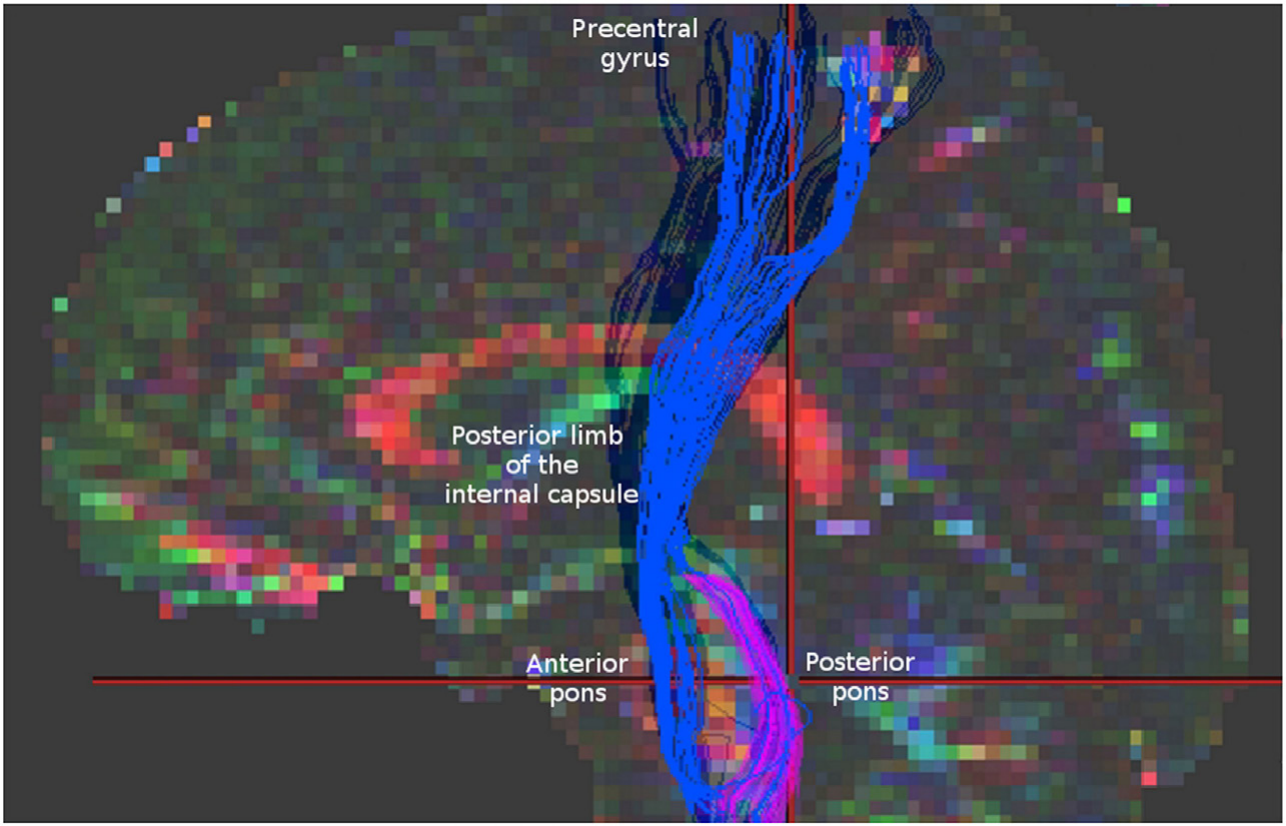
Blue fibers correspond to corticospinal tract and alternate motor fibers (AMF) reconstructed according to Lindenberg et al. (4). Magenta fibers represent spinothalamic tract fibers obtained according to Dubois et al. (9) for the same data. Note a similar appearance of these two reconstructions in the region between the pons and the thalamus. For comparison check the Figure 1 in Lindenberg et al. (4) presenting original reconstructions of AMF.

Thus, knowing that thalamus has relatively low FA values, it seems possible that at least some STT and medial lemniscus fibers could be misinterpreted as AMF in the region between the pons and the thalamus. These sensory pathways can be computationally fused with the CST and/or AMF at the level of the thalamus and then falsely tracked along their trajectories up to the cortex. Importantly, since this fusing occurs between the pons and the thalamus, no ROI above the thalamus level, including the one used in the precentral gyrus, could prevent the occurrence of this artifact. Theoretically, one may consider putting additional ROIs in thalamus and/or in brain stem to make fiber selection in a more rigorous way. However, this would not be an easy task either because (1) there is no established standard for segmenting thalamus into distinct nuclei. Moreover, at the thalamic level reconstructed fibers projecting to sensory and motor cortical areas exhibit a substantial overlap (11); (2) brain stem has a very dense fiber architecture which is difficult to resolve using standard DWI protocols (12). Further investigation of such possible sensory component of the AMF would require using more elaborated non-tensor diffusion models, such as Q-ball imaging, constrained spherical deconvolution imaging, or kurtosis imaging, to disentangle the tightly packed fibers at the pontine level and to avoid artificial fiber fusing at the level of the thalamus. Recently, it was also suggested that adequate tracking of human sensorimotor tracks may be achieved using probabilistic tractography with adaptive thresholding (13). However, comparison of such approaches was beyond the scope of this short communication as our main goal was to bring more attention to the fact that AMFs may also have an additional sensory counterpart which should not be neglected when interpreting results in neurorehabilitation studies.

Such sensory interpretation is interesting both because AMFs were demonstrated to be clinically relevant for motor stroke recovery (2, 4, 6, 8) and because of the importance of the sensory function for motor recovery (14). The exact interpretation of sensorimotor integration in motor rehabilitation still remains challenging (15). From a physiological point of view, it has been clearly shown that motor and somatosensory systems are functioning in a tight communication and that somatosensory afferentation has a great impact on motor behavior and *vice versa* (16, 17). Clinical studies as well reported a correlation between sensory and motor outcomes (18, 19) and proved that additional sensory stimulation and training of the affected hand may be beneficial for motor rehabilitation (18, 20) and the other way around—that anesthesia of the unaffected limb may improve motor performance of the affected one (19). At the same time, neuroimaging findings for the role of sensory pathways in movement restoration are still scarce. To our knowledge, there are only few works demonstrating a possible involvement of thalamocortical fibers (21, 22) and sensory regions of the corpus callosum (23) in stroke motor recovery. Therefore, if AMF indeed includes a sensory component then its relation to motor recovery may present an important neuroimaging demonstration of the role of the sensory pathways for motor recovery in stroke. Further functional verification of the AMF sensory component would require a demonstration of a correlation of their integrity with sensory function and its dynamical changes in recovering stroke patients. In addition, it would be important to investigate a link between AMF and the efficacy of the sensory approaches for motor stroke rehabilitation such as proprioceptive training (24) or somatosensory nerve stimulation (20).

To sum up, in this paper we aimed at bringing attention to a possible sensory interpretation of the so-called AMF—a new structural reserve component for motor stroke recovery. Considering limitations of the DTI approach, more elaborate and rigorous investigation of the topic is warranted. This can be done for instance using non-tensor diffusion models and probabilistic tractography, which might help delineating the fiber composition at the pontine level. In addition, such possible new interpretation of AMF might provide a new outlook on the importance of sensory pathways in motor recovery after stroke.

## Author Contributions

SK and MN suggested the idea of the work, interpreted data for the work, wrote the manuscript, made final improvement, and agreed to be accountable for all aspects of the work. VN and LD interpreted data for the work, critically revised the manuscript, made final approvement, and agreed to be accountable for all aspects of the work.

## Conflict of Interest Statement

The authors declare that the research was conducted in the absence of any commercial or financial relationships that could be construed as a potential conflict of interest.
